# A foodborne acute gastroenteritis outbreak caused by GII.P16-GII.2 norovirus in a boarding high school, Beijing, China: a case–control study

**DOI:** 10.1186/s13104-018-3532-1

**Published:** 2018-07-03

**Authors:** Xin-hui Guo, Zhen Kan, Bai-wei Liu, Li-li Li

**Affiliations:** 1 Fangshan Center for Disease Control and Prevention, Fangshan, Beijing, China; 2 Beijing Center for Disease Control and Prevention, Beijing, China

**Keywords:** Norovirus, Outbreak, GII.P16-GII.2, Foodborne

## Abstract

**Objective:**

In December 2017, an acute gastroenteritis outbreak involving 61 students occurred in a boarding high school in Beijing, China. We conducted an outbreak investigation immediately in order to determine the cause of this outbreak and provide effective control measures.

**Results:**

The laboratory inspection showed that this outbreak was caused by GII.P16-GII.2 norovirus. Risk factor analysis indicated that the lunch provided by Cafeteria 1 on Dec 12 might be the risk factor of the outbreak with an odds ratio (OR) of 3.800 (95% confidence interval [CI] 1.089–13.258). Additionally, a tray line server of Cafeteria 1 was found to have gastro-enteral symptoms recently. Based on the clinical symptoms and epidemiology investigation, the symptomatic server was considered to be the possible source of infection.

## Introduction

Norovirus (NoV) is an important viral pathogen of acute gastroenteritis (AGE) [1–3]. It is estimated to be associated with about a fifth of all cases of AGE worldwide [4]. NoV is also the leading cause of epidemic gastroenteritis [5]. According to the Nation Outbreak Reporting System, NoV is responsible for 68% of the AGE outbreaks occurred in the United States during 2009–2010 [6]. In China, most of the norovirus outbreaks occurred in schools [7]. On December 14, 2017, we were notified of an AGE outbreak occurred in a boarding high school, Beijing, China. This study was conducted immediately in order to determine the causative pathogen and source of infection, and eventually, to provide effective control measures.

## Main text

### Methods

#### Epidemiological investigation

Case definitions: Probable cases were defined as patients who developed symptoms of vomiting ≥ 1 time/day and/or diarrhea ≥ 3 times/day in this school from Dec 13 to Dec 16, 2017, among which patients tested positive for noroviruses by real-time reverse transcription PCR were defined as laboratory-confirmed cases. All of the cases in this outbreak were interviewed via telephone on age, sex, occupation, class, dormitory, symptom onset time and clinical symptoms.

In order to find out the cause of this outbreak, a case–control study was conducted by using a semi-structured questionnaire to interview the subjects on suspected risk factors. In the case–control study, cases were defined as patients who developed symptoms of acute gastroenteritis (i.e. vomiting ≥ 1 time/day or diarrhea ≥ 3 times/day) on Dec 13 and Dec 14, 2017 in this school. Age- and class-matched controls (1:1) were selected from the remaining students. The suspected risk factors included the cafeterias they had gone to from Dec 11, 2017 to Dec 12, 2017, different kinds of water they had drunk, sanitation practices, and auxiliary classes.

#### Food and water investigation

Information on food and drinking water supply was obtained from the vice principal of this school. Recent health condition of the cafeteria staff was obtained via face-to-face or telephone interviews.

#### Sample collection

Samples of stool, vomitus or rectal swabs were collected from the patients within 5 days post symptoms onset. Rectal swabs or stool samples were taken from the staff of cafeterias. Surface swabs were taken from the environment of kitchens. Stool and vomitus samples were directly placed in sterilized sealed containers without dilution. Rectal and surface swabs were inserted into tubes containing 2 mL sterilized phosphate buffer solution (PBS). All of the samples were placed on ice and transferred as soon as possible to the laboratory of local CDC for pathogen analysis.

#### Laboratory examination

Specimens from the patients were tested for the presence of common pathogenic bacteria and viruses. The other samples were only tested for the presence of noroviruses. Common pathogenic bacteria including diarrheagenic *Escherichia coli*, *salmonella*, *Shigella*, *Vibrio parahaemolyticus*, *Campylobacter jejuni* and *Yersinia pseudotuberculosis* were identified via bacterial culture according to the technical procedures for Diarrheal Pathogenic Spectrum Surveillance formulated by China Center for Disease Control and Prevention. Common pathogenic viruses including norovirus and rotavirus were detected with the method of real-time reverse transcription PCR. All positive PCR products of patient specimens were sequenced and compared with reference strains to determine the genotypes.

#### Statistics analysis

Binary data was analyzed by Chi square test or Fisher’s exact test. Ordinal categorical data was analyzed by Cochran–Armitage test for trend. Odds ratios and 95% confidence intervals were calculated for suspected risk factors in relation to the disease of interest. Statistical analyses were performed with SPSS 22.0. Differences were considered significant if *p* value was < 0.05.

### Results

#### Clinical symptoms

All of the cases were not serious in clinical symptoms and recovered within 4 days post the onset date. The most common signs and symptoms were vomiting (88.14%), nausea (86.44%), abdominal pain (83.05%), fever (38.98%) and diarrhea (25.42%).

#### Illness onset time

**Fig. 1:**
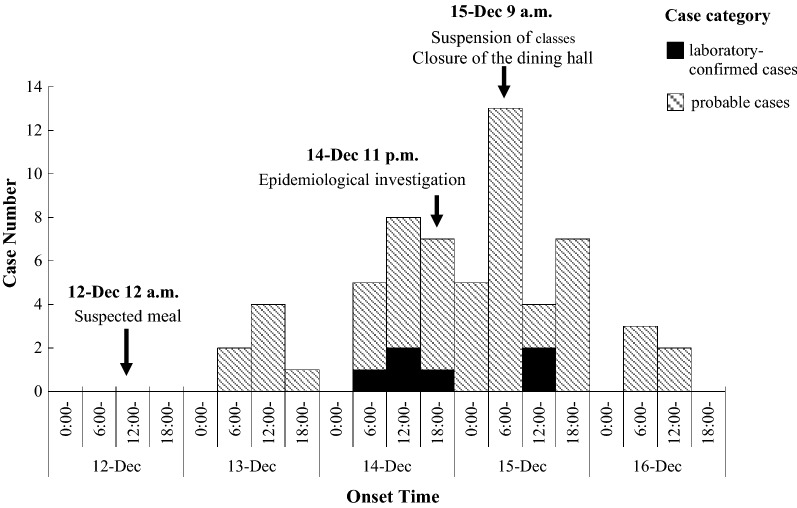
Date and time of onset of illness among cases (n = 61)

The first case developed gastrointestinal symptoms at 7:00 a.m. on Dec 13, 2017. As of Dec 16, the onset date of the last case, a total of 61 students were involved in this outbreak with an attack rate of 3.80% (61/1606). The number of new cases reached peak on 15-Dec. Figure 1 shows the distribution of cases by the date and time of symptom onset.

#### Age, sex, class and dormitory

**Fig. 2:**
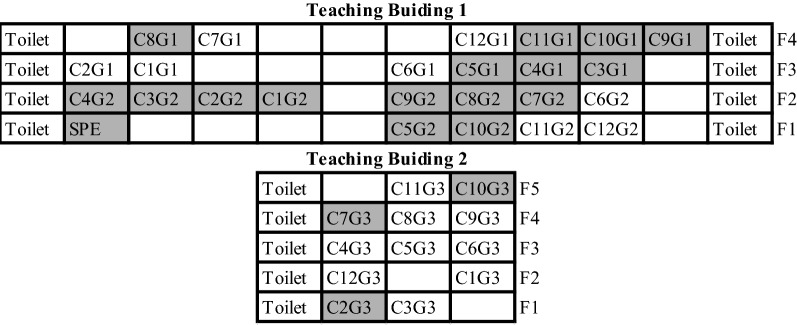
Spatial distribution of the 61 cases among the 37 classes in the school. *C* Class, *G* Grade, *SPE* Special class. Cells filled grey indicate that at least one case was identified from this class

All of the cases were students. The median age of them was 16 (range 14–17) years and the male–female ratio was 2.69. There were 37 classes (spatial distribution presented in Fig. 2) in this high school, among which 20 classes were found to have cases. Most of the cases (96.72%, 59/61) were boarders who live in dormitories from Monday to Friday. The 59 boarding cases scattered among 40 dormitories without obvious evidence of clustering.

#### Case–control study

A total of 27 cases and 27 non-cases were selected for the case–control study. All of them have completed the questionnaire. The results of statistics analyses were summarized in Table 1. Among the suspected risk factors, only the lunch on 12-Dec was found to be significantly associated with the disease (χ^2^ = 4.608, p = 0.032, OR = 3.800, 95% CI = 1.089–13.258).

**Table 1:** Results of statistical analysis for suspected risk factors

Suspected risk factors	Cases	Controls	χ^2^	P-values	OR(95% CI)
12-Dec supper	20	20			
Cafeteria 1	10	10	0.000	1.000	1.000 (0.290–3.454)
Cafeteria 2	10	10			1.000
12-Dec lunch	24	26			
Cafeteria 1	19	13	4.608	0.032*	3.800 (1.089–13.258)
Cafeteria 2	5	13			1.000
12-Dec breakfast	19	20			
Cafeteria 1	9	9	–	1.000^a^	1.100 (0.312–3.877)
Cafeteria 2	10	11			1.000
11-Dec supper	19	18			
Cafeteria 1	14	13	–	1.000^a^	1.077 (0.252–4.597)
Cafeteria 2	5	5			1.000
11-Dec lunch	25	26			
Cafeteria 1	10	11	0.028	0.867	0.909 (0.298–2.775)
Cafeteria 2	15	15			1.000
11-Dec breakfast	20	18			
Cafeteria 1	12	7	–	0.330^a^	2.357 (0.640–8.677)
Cafeteria 2	8	11			1.000
Barreled water	27	27			
Yes	20	24	1.964	0.161	0.357 (0.082–1.564)
No	7	3			1.000
Boiled water	27	27			
Yes	8	9	0.086	0.770	0.842 (0.267–2.660)
No	19	18			1.000
Wash hands after toilet	26	27			
Never	0	0	0.146	0.702^b^	–
Sometimes	2	0			
Often	6	12			
Always	18	15			
Wash hands before meal	26	27			
Never	0	4	0.324	0.569^b^	–
Sometimes	11	7			
Often	9	10			
Always	6	6			
Like cold/uncooked food	26	27			
Never	7	7	0.107	0.744^b^	–
Sometimes	9	10			
Often	7	9			
Always	3	1			
Auxiliary class	27	27			
Yes	13	17	1.200	0.273	1.831 (0.618–5.425)
No	14	10			1.000

* Statistically significant

^a^ Fisher’s exact test

^b^ Cochran–Armitage test for trend

#### Cafeterias and drinking water investigation

This school had 2 cafeterias (hereinafter referred to as Cafeteria 1 and Cafeteria 2) providing various food for students. The cafeterias were completely independent in both staff and food raw materials.

There were 27 and 17 employees working for Cafeteria 1 and 2, respectively. One of the tray line servers of Cafeteria 1 reported that, she was experiencing mild diarrhea recently however she could not recall the specific onset date. She kept working until 15-Dec, when the cafeterias were suspended.

Barreled purified water of a certain brand was provided for all students and teachers via a water dispenser in each classroom and office. In addition, there was a boiler in the school providing living and drinking water for students and teachers.

#### Laboratory inspection

A total of 34 samples were collected in accordance with the local Surveillance Program for the Acute Gastroenteritis Outbreak, including 10 stool samples from the patients, 15 rectal swabs from the cafeteria staff (the symptomatic worker was not included; she denied our request of sampling), and 9 surface swabs from the kitchens. Laboratory inspection results showed that, the specimens of 6 patients were positive for GII norovirus, but negative for the other pathogens. Sequencing and comparison of these specimens suggested that, the genotype of the pathogenic virus was GII.P16-GII.2 norovirus. The other samples were negative for any of the pathogens that had been tested.

#### Control measures

The cafeterias were closed on Dec 15, 2017. Thorough disinfection was done for the cafeterias and health education was conducted among food handlers. The tray line server with gastroenteritis symptoms was allowed to stay at home until 3 days after her symptoms completely disappeared. The classrooms and dorms were disinfected. Classes were suspended from Dec 15 to Dec 19.

### Discussion

Norovirus (NoV) infection in human is characterized by an acute onset of vomiting and/or diarrhea [8]. The results of the present investigation suggested that vomiting is the most common symptom in the current outbreak of acute gastroenteritis (AGE), which is similar to the results of most outbreak studies conducted in China [9, 10].

The average incubation period of NoV has been estimated to be 24–48 h [11]. Taking this into consideration, the epidemic curve of the present outbreak indicated a mixed epidemic involving both a common source epidemic and secondary propagated spread to other individuals.

Cases of the present outbreak were all students. The classrooms of them involved almost every floor in the two teaching buildings of this school, indicating that the source of infection might not lie in the teaching building. Likewise, it is unlikely to be the dormitory-related factors that have caused the outbreak, because there is no evidence of clustering among dorms.

In order to find out the source of infection, diet-, water-, sanitation- and auxiliary class-related risk factors were inspected in the case–control study. Only the cases with an onset date of the first 2 days were included in the analysis, in order to avoid the influence of the secondary person-to-person transmission. The statistical results showed that, the lunch provided by Cafeteria 1 on Dec 12 might be the risk factor of the outbreak with an odds ratio of 3.800 (95% confidence interval, 1.089–13.258).

Additionally, a tray line server of Cafeteria 1 was found to have gastro-enteral symptoms recently. Based on the clinical symptoms and epidemiology investigation, this server was considered to be the possible source of infection.

After the symptomatic tray line server was temporally removed from her post and comprehensive control measures were taken, the number of cases decreased rapidly. This observation offered another evidence for the deduction that the server was the potential source of this outbreak.

### Conclusion

In conclusion, this outbreak of GII.P16-GII.2 norovirus might be a foodborne event possibly caused by an infected tray line server. Proper food handler training should be strongly recommended in schools to prevent foodborne infectious diseases.

## Limitations

There were several limitations in our analysis, including (1) the recall bias existing in this kind of retrospective study; (2) the lack of laboratory evidences for the symptomatic tray line server; (3) it is difficult for the cases and controls to recall the specific tray line server they get food from on Dec 12.

## Abbreviations

CDC: Center for Disease Control and Prevention
AGE: acute gastroenteritis
NoV: norovirus
PCR: polymerase chain reaction

## References

[1] Cheng HY, Hung MN, Chen WC, Lo YC, Su YS, Wei HY (2017). Ice-associated norovirus outbreak predominantly caused by GII.17 in Taiwan, 2015. BMC Public Health.

[2] Payne DC, Vinjé J, Szilagyi PG, Edwards KM, Staat MA, Weinberg GA (2013). Norovirus and medically attended gastroenteritis in US children. New Engl J Med.

[3] Neo FJX, Loh JJP, Ting P, Wei XY, Gao CQH, Lee VJM (2017). Outbreak of caliciviruses in the Singapore military, 2015. BMC Infect Dis.

[4] Ahmed SM, Hall AJ, Robinson AE, Verhoef L, Premkumar P, Parashar UD (2014). Global prevalence of norovirus in cases of gastroenteritis: a systematic review and meta-analysis. Lancet Infect Dis.

[5] Patel MM, Hall AJ, Vinje J, Parashar UD (2009). Noroviruses: a comprehensive review. J Clin Virol.

[6] Hall AJ, Wikswo ME, Manikonda K, Roberts VA, Yoder JS, Gould LH (2013). Acute gastroenteritis surveillance through the national outbreak reporting system, United States. Emerg Infect Dis.

[7] Zhou N, Zhang H, Lin X, Hou P, Wang S, Tao Z (2015). A waterborne norovirus gastroenteritis outbreak in a school, eastern China. Epidemiol Infect.

[8] Shi C, Feng WH, Shi P, Ai J, Guan HX, Sha D (2016). An acute gastroenteritis outbreak caused by GII.17 norovirus in Jiangsu Province, China. Int J Infect Dis.

[9] Zha RS, Xia Y, Xue-Rong YA, Hang H, Liu C, Jian LI (2014). Characteristic of 14 norovirus gastroenteritis outbreaks in Suzhou City. Jiangsu J Prev Med..

[10] Tian J, Lili LI, Shi L, Dong R, Kan Z, Cui L (2017). Epidemiological survey of an outbreak of acute gastroenteritis caused by norovirus G Type I.6 in one high school in Fangshan district, Beijing. J Prev Med Inf..

[11] Patel MM, Hall AJ, Vinje J, Parashar UD (2009). Noroviruses: a comprehensive review. J Clin Virol..

[12] Liao QH, Ran L, Jin M, Cui SH, Yuan J, Ma HL (2016). Guidelines on outbreak investigation, prevention and control of norovirus infection (2015). Zhonghua Yu Fang Yi Xue Za Zhi.

